# ERCC1 expression affects outcome in metastatic pancreatic carcinoma treated with FOLFIRINOX: A single institution analysis

**DOI:** 10.18632/oncotarget.9063

**Published:** 2016-04-27

**Authors:** Antonia Strippoli, Sabrina Rossi, Maurizio Martini, Michele Basso, Ettore D'Argento, Giovanni Schinzari, Rosalba Barile, Alessandra Cassano, Carlo Barone

**Affiliations:** ^1^ Department of Medical Oncology, Catholic University of Sacred Heart, 00168 Rome, Italy; ^2^ Department of Pathology, Catholic University of Sacred Heart, 00168 Rome, Italy

**Keywords:** metastatic pancreatic cancer, oxaliplatin sensitivity, ERCC1, survival, response to treatment

## Abstract

**Introduction:**

No clinically useful predictive factor has been yet identified for treatment of metastatic pancreatic cancer (mPC). It is noteworthy that FOLFIRINOX, despite its high toxicity, is effective only in some patients. We retrospectively analyzed expression of excision repair cross-complementing group-1 (ERCC1) - involved in the repair of platinum induced damage - in patients affected by mPC treated with FOLFIRINOX in order to evaluate its predictive role.

**Results:**

FOLFIRINOX resulted more effective in patients with normal ERCC1 levels than in those with ERCC1 hyper-expression. Median progression free survival (PFS) was 11 vs. 4 months (HR 0.26; 95% CI 0.14-0.50; p<.0001), median overall survival (OS) 16 vs. 8 months (HR 0.23; 95% CI 0.12-0.46; p<.0001) and disease control rate (DCR) 93% vs. 50% (p=0.00006). The advantage was confirmed at univariate and multivariate analysis.

**Patients and Methods:**

71 patients with histologically proven mPC and treated with FOLFIRINOX as first-line therapy were considered eligible. mRNA ERCC1 expression was determined using RT-PCR analysis.

**Discussion:**

ERCC1 might be an effective predictor of response to FOLFIRINOX in mPC. Patients overexpressing ERCC1 should be excluded by this often toxic therapy and referred to an alternative treatment.

## INTRODUCTION

Metastatic pancreatic carcinoma (mPC) is the fourth leading cause of cancer-related death in North America and Europe [1,2]. More than 80% of patients have unresectable locally advanced or metastatic disease at diagnosis, therefore they are not eligible for potentially curative surgery [3]. Among patients with early stage disease who undergo surgical resection, more than 80% relapse within two years [4,5]. Prognosis for these patients is extremely poor and they are mainly candidate to chemotherapy, but the impact of standard therapy is minimal [6,7]. Newer regimens as FOLFIRINOX (oxaliplatin, irinotecan, fluorouracil and leucovorin) or gemcitabine/nab-paclitaxel have shown improved efficacy when compared to gemcitabine alone, and they are currently considered standard treatment for patients with good performance status [8,9].

The main reason for failure of chemotherapy is drug-resistance. Although resistance to chemotherapy is multifactorial, DNA repair seems to play a key role in resistance to oxaliplatin. In fact, oxaliplatin can produce inter- and intra-strand platinum-DNA cross-links that, in turn, lead to the inhibition of DNA replication and transcription. Excision repair cross-complementing 1 (ERCC1) is a gene encoding a protein of the nucleotide excision repair (NER) complex, which includes a group of proteins that are able to repair the DNA damage induced by adduct-forming agents, such as platinum-derivatives [10]. A functional ERCC1 is essential for repairing platinum–DNA adducts and it is involved in drug-sensitivity *in-vitro* [11,12]. In the past years, the role of ERCC1 as predictive factor in patients affected by gastrointestinal cancers and treated with oxaliplatin-based chemotherapy has been extensively investigated, but results are controversial. In a recent meta-analysis, Ma et al. evaluated the association between ERCC1 polymorphisms and clinical outcome of oxaliplatin-based chemotherapy in 22 studies including gastric and colorectal cancer [13]. For the *ERCC1* rs11615 polymorphism, the T allele was associated with a reduced response to chemotherapy in Asians patients and in gastric cancer population (p< .05) as well with a significant shorter PFS and OS in all patients (PFS: HR =1.22, p< .001; OS: HR=1.12, p< .001).

In the present study we retrospectively investigated whether the expression of ERCC1 may be a prognostic or predictive factor in patients affected by mPC and treated with FOLFIRINOX combination therapy.

## RESULTS

### Patient characteristics and treatments

Seventy-one out of 82 patients with histologically proven diagnosis of mPC treated in our center between August 2010 and October 2014 were considered eligible. Patients were divided into two groups, according to the established cutoff value of ERCC1: those with mPC carrying ERCC1 overexpression (ERCC1+) and patients with normal ERCC1 levels (ERCC1-). In ERCC1+ group most of patients were male (70%), in ERCC1- group male and female were balanced (49% and 51%, respectively); median age at first diagnosis of mPC was 60 years (range 44-78) and 63 years (range 40-81) in the two groups, respectively. Performance status at diagnosis was better in ERCC1+ patients (ECOG-PS =0 in 53% of cases; ECOG-PS =1 in 47% of cases) than in ERCC1- group (ECOG-PS =0 in 39% of cases; ECOG-PS =1 in 61% of cases). Only six out of 71 patients (8.4%) underwent surgery and received an adjuvant treatment with gemcitabine (1000 mg/m^2^, on days 1-8-15 of a 21 days cycle for 6 courses); only one out of 6 patients received adjuvant radiotherapy. Primary site was the pancreatic head in 53% and 56% ERCC1+ and ERCC1- patients and pancreatic body/tale in 47% and 44% of patients in the two groups, respectively. The ERCC1- cohort included more patients with lymph nodes-limited disease (32 vs 10%), although the two groups are quite balanced considering patients with multiple metastatic sites (54% and 63% in ERCC1- and +, respectively). FOLFIRINOX was started at full doses in 57% of ERCC1+ group and 61% of ERCC1- patients, whereas it was started at 75% of the full dose in 43% and 39% of patients in the two cohorts, respectively. Twelve courses of chemotherapy were completed more frequently in ERCC1- patients (80%) than in ERCC1+ patients (23%). Forty out of 71 patients underwent a second-line treatment: 25 out of 41 (61%) patients in ERCC1- group and 15 out of 30 (50%) in ERCC1+ group. In most cases, a monochemotherapy with gemcitabine (47.5%) was administered; otherwise patients received nab-paclitaxel (27.5%) or oral capecitabine (25%). In ERCC1- group, 60% of patients received gemcitabine, 36% nab-paclitaxel and 4% oral capecitabine. In ERCC1+ patients, oral capecitabine was administered in 60% of cases, gemcitabine in 27% and nab-paclitaxel in 13%. Patients' characteristics are summarized in Table 1.

**Table 1:** Patients' characteristics

	ERCC1 + (n. 30)	ERCC1 − (n. 41)	P value
**Sex**			
- Male	21/30 (70%)	20/41 (49%)	0.12
- Female	9/30 (30%)	21/41 (51%)	
**Median age at diagnosis**	60 (range 44-78)	63 (range 40-81)	−-----
**Performance status at diagnosis**			
- 0	16/30 (53%)	16/41 (39%)	0.33
- 1	14/30 (47%)	25/41 (61%)	
**Primary site**			
- Head	16/30 (53%)	23/41 (56%)	0.99
- Body/tail	14/30 (47%)	18/41 (44%)	
**Site of metastases**			
- Lymph nodes	3/30 (10%)	13/41 (32%)	
- Liver	8/30 (27%)	6/41 (14%)	0.08
- Multiple sites	19/30 (63%)	22/41 (54%)	
**Previous adjuvant treatment**	1/30 (3%)	5/41 (12%)	0.37
**FOLFIRINOX dose-intensity**			
- Full dose	17/30 (57%)	25/41 (61%)	0.90
- Lower dose (75%)	13/30 (43%)	16/41 (39%)	
**FOLFIRINOX x12 cycles**			
- Completed	7/30 (23%)	33/41 (80%)	<0.0001
- Not completed	23/30 (77%)	8/41 (20%)	
**SECOND-LINE THERAPY**	15/30 (50%)	25/41 (61%)	.47
- Gemcitabine	4/15 (27%)	15/25 (60%)	
- Nab-paclitaxel	2/15 (13%)	9/25 (36%)	.0001
- Capecitabine	9/15 (60%)	1/25 (4%)	

At a median follow-up of 36 months, 63 deaths (89%) had occurred and only one patient with normal expression of ERCC1 (1%) did not experience progressive disease during or after FOLFIRINOX treatment. In the whole cohort of patients, median PFS was 7 months and median OS 12 months. No differences in PFS and OS were found when primary site was pancreatic head or pancreatic body/tale (PFS: 8 vs. 7 months, HR 0.92, 95% CI 0.58-1.47, p= .71; OS: 12 vs. 13 months; HR 1.14, 95% CI 0.70–1.87, p= 0.57). There was no significant relationship between ERCC1 expression and sex or site of primary tumor (pancreatic head or body/tale) or metastatic sites (lymph nodes, liver or multiple sites) or previous adjuvant treatment.

### Response and survival

**Figure 1:**
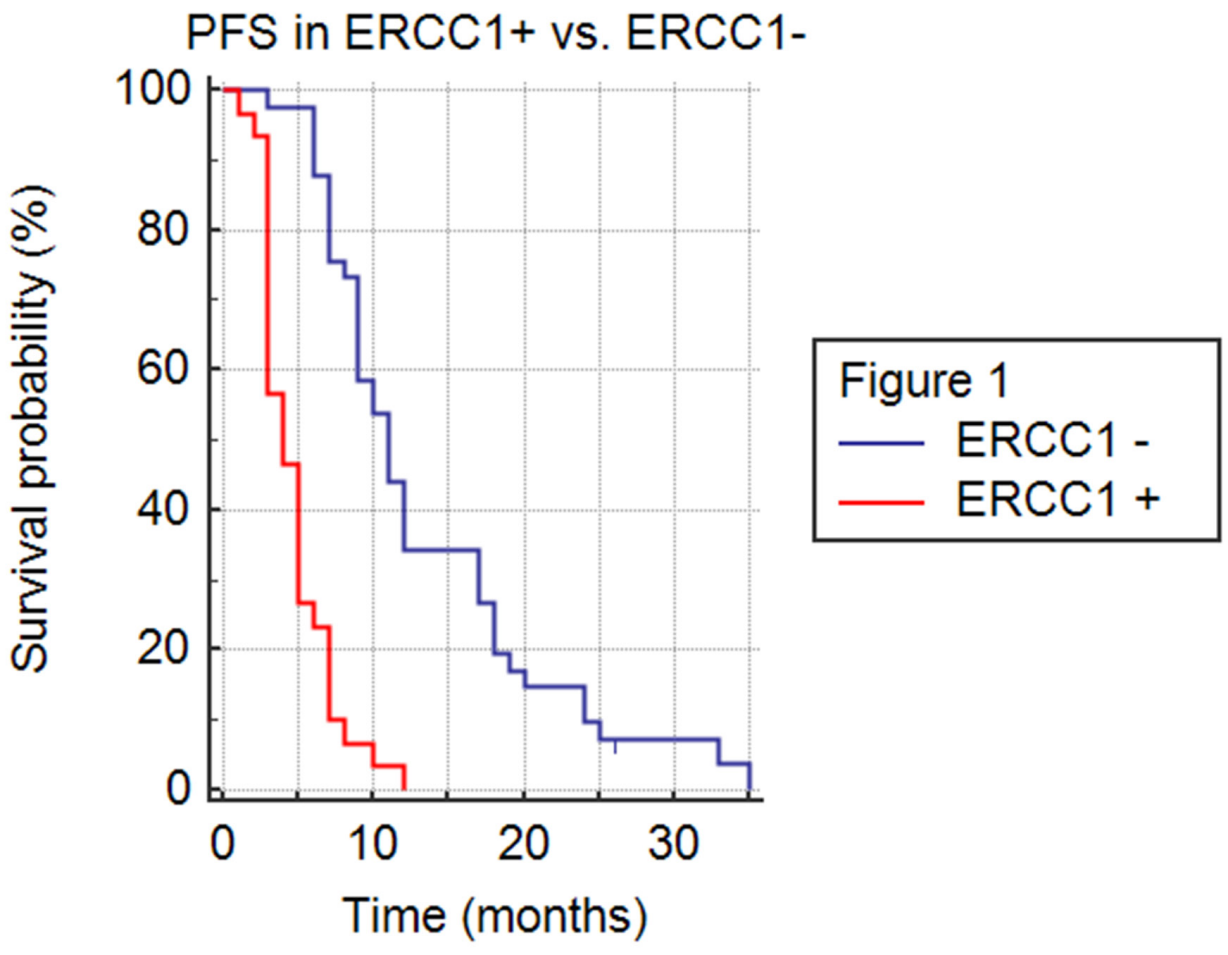
Progression free survival (PFS) in ERCC1+ versus ERCC1- population

**Figure 2:**
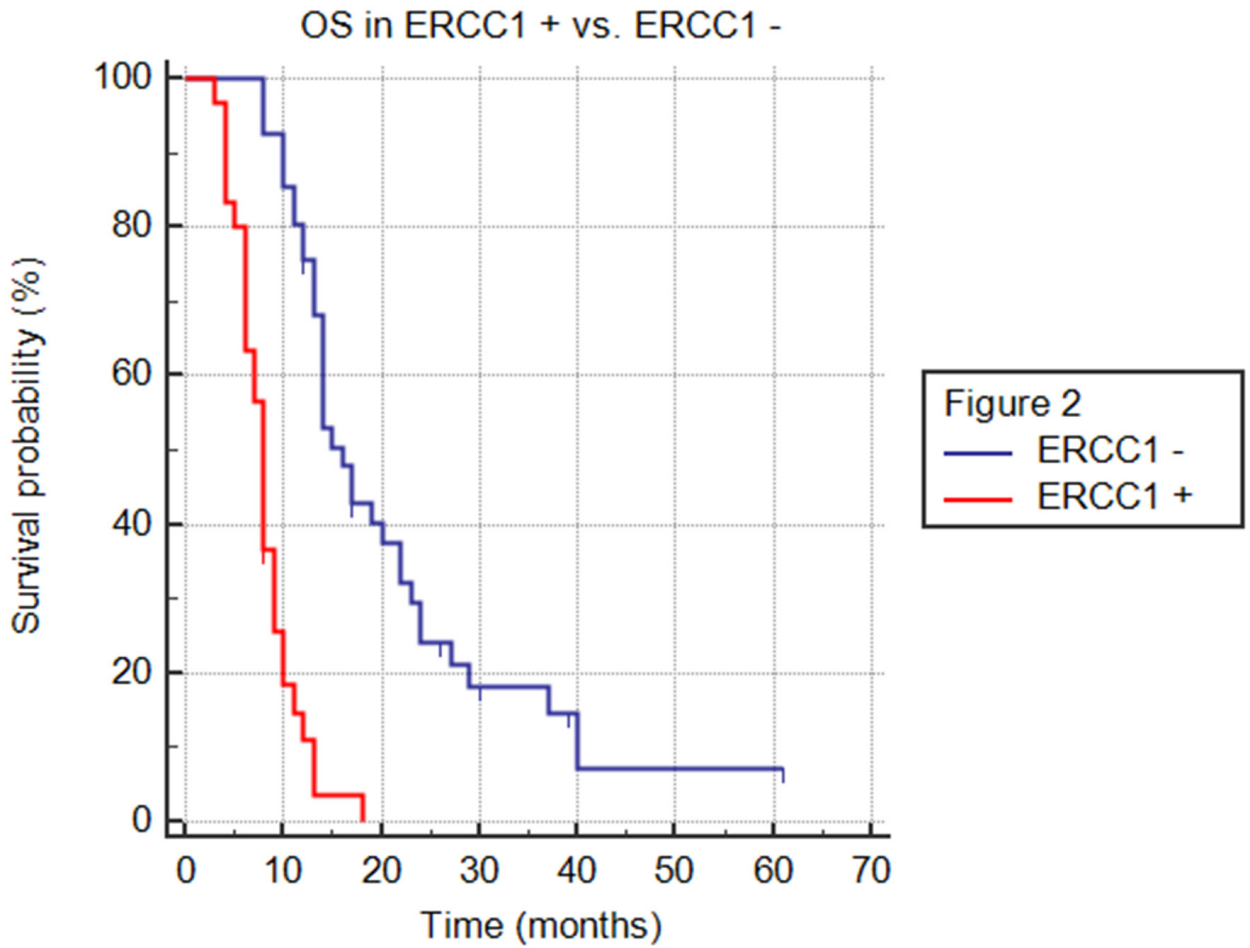
Overall survival (OS) in ERCC1+ versus ERCC1- population

The efficacy of FOLFIRINOX was higher in 41 patients with normal ERCC1 levels than in 30 patients with ERCC1 hyper-expression; PFS was 11 vs. 4 months (HR 0.26; 95% CI 0.14-0.50; p<.0001), OS was 16 vs. 8 months (HR 0.23; 95% CI 0.12-0.46; p<.0001) and DCR was 93% and 50% (p= .00006), respectively (Figures 1–2 and Table 2). Patients with pancreatic head adenocarcinoma and normal ERCC1 levels had a significant survival benefit when compared to those hyper-expressing ERCC1 (PFS: 11 vs. 3 months, HR 0.30, 95% CI 0.13-0.70, p< .0001; OS: 16 vs. 7 months; HR 0.23, 95% CI 0.09–0.57, p<.0001) and a better DCR (91% vs. 44%; p= .003). Similar results were obtained comparing patients with pancreatic body/tale adenocarcinoma with normal levels of ERCC1 to those with higher expression (PFS: 11 vs. 5 months, HR 0.24, 95% CI 0.09-0.62, p< .0001; OS: 14 vs. 8 months; HR 0.25, 95% CI 0.09–0.66, p<.0001; DCR 94% vs. 57%; p= .03).

**Table 2:** Disease control rate (DCR) in all subgroups of ERCC1+ and ERCC1- patients

	*CR+PR+SD = DCR*	*P value*
**ERCC1 + mPA**	15/30 (50%)	0.00006
**ERCC1 – mPA**	38/41 (93%)	
**Pancreatic head ERCC1 +**	7/16 (44%)	0.003
**Pancreatic head ERCC1 −**	21/23 (91%)	
**Pancreatic body/tale ERCC1 +**	8/14 (57%)	0.03
**Pancreatic body/tale ERCC1 −**	17/18 (94%)	
**ERCC1 + mPA (lymph nodes-limited disease)**	2/3 (67%)	0.18
**ERCC1- mPA (lymph nodes-limited metastasis)**	13/13 (100%)	
**ERCC1 + mPA (liver-limited metastasis)**	5/8 (63%)	0.58
**ERCC1- mPA (liver-limited metastasis)**	5/6 (83%)	
**ERCC1 + mPA (pluri-metastatic)**	8/19 (42%)	0.002
**ERCC1 − mPA (pluri-metastatic)**	20/22 (91%)	
**ERCC1 + mPA (Liver-limited + pluri-metastatic)**	14/27 (52%)	0.003
**ERCC1 − mPA (Liver-limited + pluri-metastatic)**	25/28 (89%)	
**ERCC1 + frail patients**	4/13 (31%)	0.01
**ERCC1 − frail patients**	13/16 (81%)	

CR, complete response; PR, partial response; SD, stable disease; mPA, metastatic pancreatic adenocarcinoma.

ERCC1+ and ERCC- cohorts were unbalanced when the number and the sites of metastases were considered (lymph nodes-limited disease, liver-limited disease or pluri-metastatic disease). Nevertheless, the advantage in terms of survival in ERCC1- patients was confirmed in all subgroups, in lymph nodes-limited disease (PFS: 12 vs. 4 months, HR 0.10, 95% CI 0.004-2.56, p< .0001; OS: 22 vs. 8 months; HR 0.16, 95% CI 0.01–2.05, p=.0006), in liver-limited disease (PFS: 11 vs. 5 months, HR 0.46, 95% CI 0.16-1.36, p= .08; OS: 14 vs. 8 months; HR 0.39, 95% CI 0.13–1.17, p= .034) and in pluri-metastatic tumors (PFS: 10 vs. 4 months, HR 0.24, 95% CI 0.11-0.55, p< .0001; OS: 14 vs. 7 months; HR 0.25, 95% CI 0.11–0.57, p<.0001). In these subgroups DCR was significantly higher only in pluri-metastatic patients (p= .002) (Table 2).

Also in frail patients who started FOLFIRINOX at 75% of the full dose, efficacy of chemotherapy was greater when normal levels of ERCC1 were expressed (PFS: 9 vs. 3 months, HR 0.32, 95% CI 0.13-0.78, p= .0001; OS: 17 vs. 8 months; HR 0.25, 95% CI 0.09–0.67, p= .0001; DCR 81% vs. 31%; p= .01).

On multivariate analysis including ERCC1 expression, performance status, site and number of metastasis, initial dose of FOLFIRINOX and number of chemotherapy courses administered, only normal ERCC1 levels (p< .0001; 95% CI: 2.82-9.38) and completion of twelve courses of chemotherapy (p= .0046; 95% CI: 0.27-0.78) were independently associated with longer PFS. The same variables were evaluated for OS outcome; the only independent prognostic variables were normal ERCC1 levels (p< .0001; 95% CI: 3.15-11.3) and completion of twelve courses of chemotherapy (p= .018; 95% CI: 0.28-0.88).

Furthermore, we evaluated the influence of ERCC1 expression in second-line therapy: median PFS in ERCC1+ and ERCC1- patients was similar and not statistically significant (2 vs. 2 months; HR 0.99; 95% CI: 0,49-1,99; p= .98).

### Adverse events and tolerability

The most frequent adverse events were myelosuppression, diarrhea, anorexia, nausea and vomiting, mucositis and paraesthesia. There was no treatment-related death both in ERCC1+ and ERCC1- population, but ten cancer-related deaths (33%) occurred in ERCC1+ cohort. Adverse events of grade 3/4 were infrequent and comparable in the two groups. Nine patients with ERCC1 normal levels (22%) and 7 patients with ERCC1 hyper-expression (23%) required dose reduction for treatment-related adverse events, while 25 ERCC1- patients (61%) and 19 ERCC1+ patients (63%) had a dose delay for myelosuppression and diarrhea. Treatment interruption due to serious adverse events occurred in four patients in the ERCC1+ cohort (13%) and in four patients with normal levels of ERCC1 (10%).

Thirty-three out of 41 ERCC1- patients (81%) and eight out of 30 ERCC1+ patients (26%) experienced an improvement of performance status after treatment with FOLFIRINOX.

## DISCUSSION

Only few studies have been aimed to evaluate prognostic and predictive factors in pancreatic cancer. In the present retrospective analysis we evaluated the role of ERCC1 in patients affected by mPC treated with FOLFIRINOX. *In-vitro* studies suggest that the repair of oxaliplatin-induced DNA damage is related to platinum salts resistance [14,15,16]. The NER pathway is mainly involved in this process, in which the endonuclease encoded by the ERCC-1 gene is the rate-limiting step [17,18,19]. Overexpression of ERCC1 has been related to resistance to platinum-based therapy in different solid tumors [20,21,22,23,24]. However, other studies have excluded any association between ERCC1 and clinical outcome after oxaliplatin-based chemotherapy [25,26]. Anyway, ERCC1 seems an independent poor prognostic marker regardless of oxaliplatin-contaning chemotherapy, as high expression is associated with reduced survival [27]. In a recent study Maithel et al. prospectively selected 95 patients who underwent pancreaticoduodenectomy for pancreatic adenocarcinoma to perform immunohistochemistry for ERCC1 expression; seventy-three out of 95 patients (77%) received adjuvant chemotherapy ± radiation after resection. [28] In these patients high ERCC1 expression was associated with shorter recurrence free survival (p = 0.03) and OS (p = 0.019). A negative predictive role for ERCC1 was suggested by Mancuso et al. who reported that high ERCC1 expression was associated with reduced survival in 160 patients with advanced pancreatic cancer treated with second-line platinum therapy [29]. In this study, median survival was significantly longer in patients with low ERCC1 levels (11.9 versus 9.9 months; p ≤ 0.05) and a trend towards a longer time to progression was also observed, whereas no difference in OS was observed in patients not treated with platinum-based chemotherapy. A retrospective study in a mixed population of 89 patients affected by non-small-cell lung cancer (n. 45), ovarian cancer (n.27) and pancreatic cancer (n.17), but treated with cisplatin-containing regimens, has shown that median survival of patients with low level of ERCC1 expression was 18 months in comparison to 12 months of those with high level of expression [30]. No statistically significant association was found between ERCC1 expression and response to therapy in the small group of patients with pancreatic cancer, in which 5 out 17 patients (29%) were treated with gemcitabine alone.

**Figure 3:**
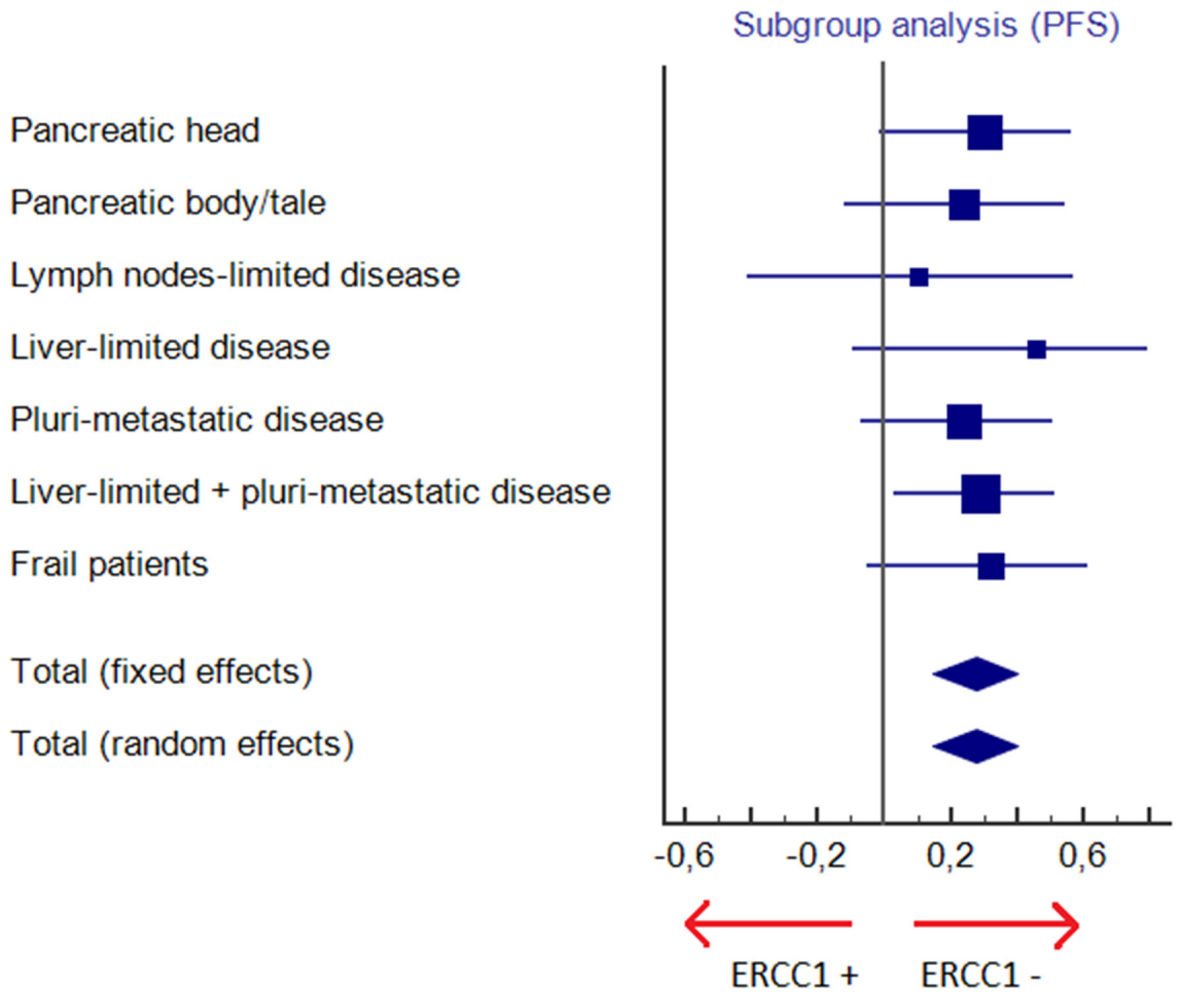
Subgroup analysis for progression free survival (PFS)

**Figure 4:**
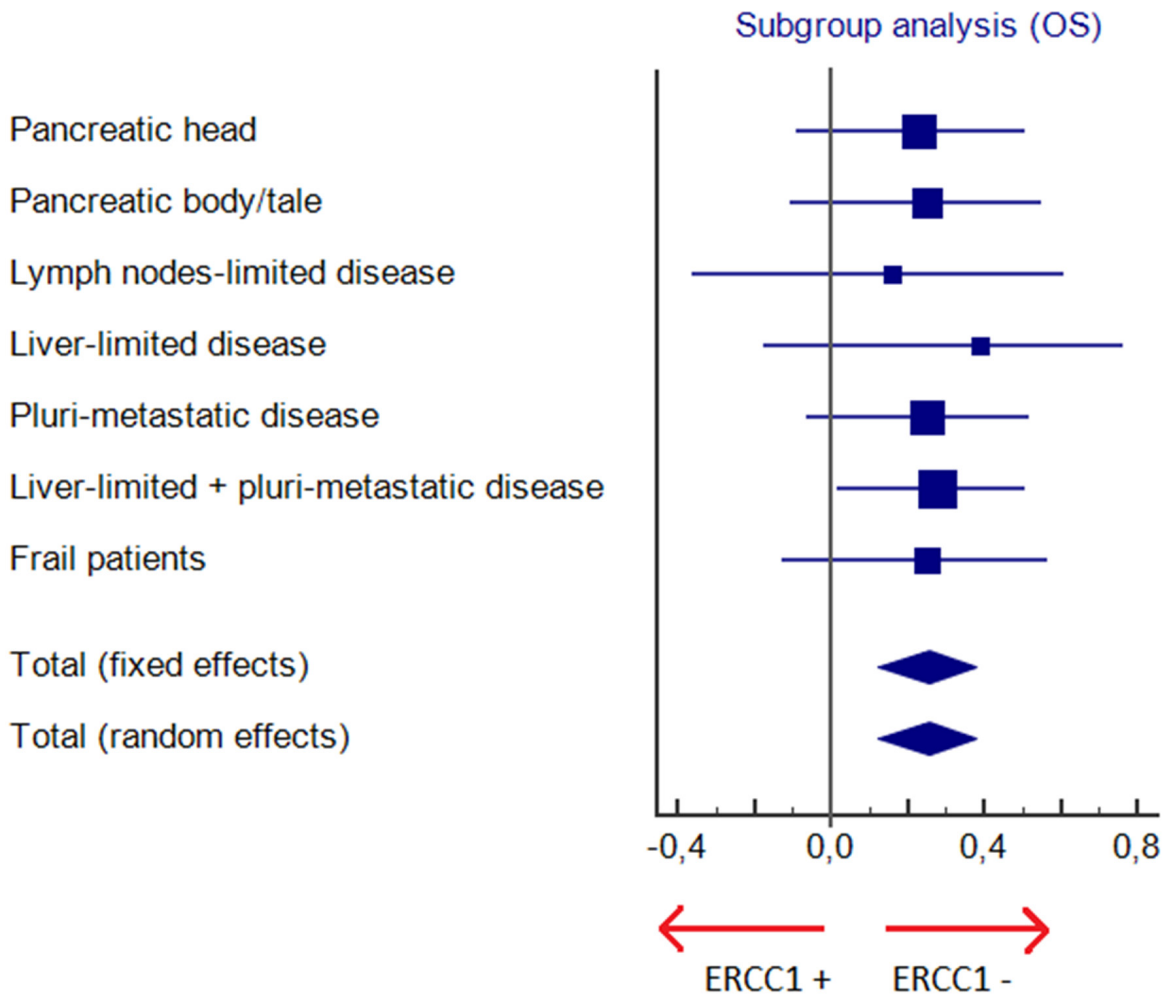
Subgroup analysis for overall survival (OS)

Therefore, the prognostic or predictive role of ERCC1 in pancreatic cancer is still unclear and the present analysis is to our knowledge the first study in a cohort of patients with mPC treated in first-line with the same oxaliplatin containing regimen. Despite the small sample size and the retrospective nature, in our study ERCC1 expression levels were strongly associated with all clinical parameters of efficacy resulting significantly lower in patients overexpressing ERCC1, in agreement with the hypothesis arising from its biochemical action. In the present study median PFS and median OS of the whole population considered are 7 months and 12 months, similar to results of the ACCORD trial in which FOLFIRINOX - compared to gemcitabine as monochemotherapy - met both the primary endpoint (OS) and secondary endpoints (PFS and quality of life, QoL). In our study, despite some unbalancement in characteristics of the two groups, in particular the higher percentage of patients with only lymph nodes involvement in ERCC1- group, ERCC1- patients achieved a 4-months advantage both in PFS and OS when compared to ERCC1+ patients, that means an increase of 50% and 30% in PFS and OS, respectively. The survival advantage in ERCC1- cohort was confirmed in all subgroups (Figures 3–4) and was independent from primary site (pancreatic head or body/tale), number and site of metastases (lymph nodes-limited disease, liver-limited disease, pluri-metastatic disease) and patients' frailty. Also DCR was higher in patients with normal levels of ERCC1 and this result was statistically significant in all subgroups, except for patients with lymph node-limited and liver-limited disease, suggesting that the prevalence of locally advanced patients in ERCC1- subgroup probably does not represent a significant bias. The greater efficacy of FOLFIRINOX in ERCC1- arm was emphasized by the larger proportion of patients in this subgroup who completed 12 courses of chemotherapy (83% in ERCC1- arm and 23% in ERCC1+ arm; p< .0001), due to a better disease control rate as well as to improved performance status. In addition, we demonstrated that no survival advantage was seen in second-line setting according to ERCC1 status, suggesting that the survival advantage in terms of OS for ERCC1- group is mainly due to FOLFIRINOX treatment.

Our results support the hypothesis that lower levels of ERCC1 may confer higher sensitivity to oxaliplatin-containing regimens in mPC; as a consequence, patients overexpressing ERCC1 should not be treated with a potentially toxic combination as FOLFIRINOX. Whether ERCC1 should be considered a prognostic or predictive factor is not clear. However, even if RT-PCR analysis to determine actual mRNA levels of ERCC1 is costly and time/labor expensive, it may be considered a useful parameter in the choice of chemotherapy combination in mPC. Nevertheless, our study has some limitations due to its retrospective nature, to the small sample size as well to inclusion criteria not so strict as in prospective clinical trials. Despite these biases, some results might be useful in the clinical practice, thus taking into account the toxicity of FOLFIRINOX and the discouraging scenario of therapeutic options in mPC, the hypothesis generated by the present study needs validation in larger randomized prospective trials.

## PATIENTS AND METHODS

### Patient selection

The study was designed as a retrospective analysis of patients (aged ≥ 18 years) with histologically proven mPC and at least one measurable metastatic lesion, treated in our center between August 2010 and October 2014. Among eighty-two treated with first-line FOLFIRINOX, 71 patients were selected based on availability of sufficient tissue for ERCC1 expression analysis. All mutational analyses on histologic samples were conducted in the laboratory of Diagnostic Molecular Pathology at the Catholic University of Sacred Heart (Rome, Italy). Additional inclusion criteria were: a) good performance status (ECOG PS = 0-1); b) first-line therapy with FOLFIRINOX (at full or modified dose on clinical judgement); c) imaging assessment (CT or PET-CT) performed at regular intervals (no longer than 3 months); d) complete informations on performance status, toxicities, dose reductions, previous adjuvant treatment. Patients were excluded in case of concomitant or prior malignancies within 5 years before starting treatment for mPC. Patients treated after December 2014 were also excluded to assure a minimum follow-up of at least one year. The study has been conducted in accordance with the rules of the local Ethics Committee and the Declaration of Helsinki. All patients provided a written consent for use of their clinical data, including molecular analyses.

### Treatments

Forty-two out of 71 patients received full-dose FOLFIRINOX as first-line therapy at full doses (oxaliplatin 85 mg/m^2^, irinotecan 180 mg/m^2^, bolus 5-fluorouracil 400 mg/m^2^, 5-fluorouracil 2400 mg/m^2^ as continuous infusion over 46 hours, leucovorin 400 mg/m^2^); the remaining 29 patients were treated with 75% of dose on clinical judgement based on patient frailty. The treatment was continued until disease progression, unacceptable toxicity or patient's withdrawal. Forty patients received a second line treatment with gemcitabine (1000 mg/m^2^ on days 1, 8, and 15 every 4 weeks) or nab-paclitaxel (125 mg/m^2^ on days 1, 8, and 15 every 4 weeks) or oral capecitabine (1000 mg/m^2^ twice daily on days 1 to 14 every 3 weeks). The clinical response to treatment was classified as complete response (CR), partial response (PR), stable disease (SD) or progressive disease (PD) according to the RECIST 1.1 criteria.[31]

### mRNA extraction and ERCC1 expression

After being deparaffined, three 10-μm slides were digested overnight at 55°C in 200 μl of TENS 1x (10mM Tris pH 7.4, 10mM EDTA, 100mM NaCl and 1% SDS) with 100 mg ml^−1^ proteinase K, and RNA was then extracted by the RNAsi mini kit (Qiagen), following the manufacturer's protocol. The quantity and quality of the RNA were assessed spectrophotometrically (E260, E260/E280 ratio, spectrum 220–320 nm; Biochrom, Cambridge, UK) and by separation on an Agilent 2100 Bioanalyzer (Palo Alto, CA, USA). RNA was treated with RQ1 RNase-Free DNase (Promega, Milan, Italy) and concentrations of samples were determined by spectrophotometer. The amplification and quantification of ERCC-1 mRNA and ACTB mRNA (taken as the internal reference gene) were performed using the iScript one-step RT–PCR kit for probes (Bio-Rad, Milan, Italy) following the manufacturer's protocol. The sequences of the primers and probes used are as follows: for ERCC-1, forward 50-GGGAATTTGGCGACGTAATTC-3′, reverse 5′-GCGGAGGCTGGAACAG-3′, probe (FAM)-5′-CACAGGTGCTCTGCCCAGCACATA-3′(TAMRA); for ACTB, forward 5′-TGAGCGCGGCTACAGCTT-3′, reverse 5′-TCCTTAATGTCAGCACGATTT-3′, probe (FAM)-5′-ACCACCACGGCCGAGCGG-3′(TAMRA). All primers were used to study intron spanning to avoid contamination with genomic DNA. Thermocycler conditions were as follows: 50°C to 10 min and 95°C for 5 min, followed by 40 cycles at 95°C for 15 min and 60°C for 35 min. The relative levels of expression of the target gene (ERCC-1), compared with the internal reference gene (ACTB), were expressed as 2-ΔCt, where ΔCt is the difference between two absolute measurements: the value of Ct (cycle threshold at which the fluorescence curve reaches an exponential) of the interest gene and the value of Ct internal reference gene (ACTB).

We determined the ERCC-1 expression on primary tumor samples while the ERCC-1 normal level was established in 20 normal pancreatic tissues of those patients underwent surgical resection for pancreatic tumor. Relative mRNA expression (tumor/normal ratio) was calculated as (ERCC-1/β-actin in tumor)/(ERCC-1/β-actin in paired normal tissue). Excision repair cross complementing group-1 mRNA expression did not show a statistically significant difference in three different measurements. We found that the median of relative ERCC-1 expression was 5.21×10^−3^(range, from 0.18 to 220.67)±45.51. This value was established as the cutoff value for ERCC-1 expression. In addition, we found that ERCC-1 mRNA expression in the pancreatic tissue of 30 healthy controls was not significantly different from the ERCC-1 expressed in normal pancreatic tissue of patients. Each assay was performed in triplicate and data were processed using the CFX96 optical system software (Bio-Rad).

### Statistical analyses

Primary endpoint was progression free survival (PFS); disease control rate (DCR) was considered as primary co-endpoint. Secondary endpoint was overall survival (OS). PFS was defined as the time from the beginning of FOLFIRINOX therapy until radiologically assessed disease progression or death for any cause. DCR was defined as the proportion of patients achieving a complete/partial response plus those achieving a stable disease. OS was defined as the time from diagnosis of metastatic disease until death for any cause or last follow-up contact. The outcome was censored if a patient had not reached survival endpoints (progression/death) at the time of last follow-up. Kaplan-Meier method and the log-rank test were used to estimate PFS and OS. Multivariate Cox regression model was used to identify the predictive effect of different variables on PFS and OS. Exact Fisher test and Chi-squared test were used to establish the significance of the association between DCR and other variables. All reported p values are two-tailed and a level of 0.05 or less was considered statistically significant. Cox proportional-hazards regression analysis was used to examine the effect of variables on survival outcomes of the study population. To remove a variable from the model, the corresponding p-value had to be >0.10.
